# Protective Effects of Simvastatin on Endotoxin-Induced Acute Kidney Injury through Activation of Tubular Epithelial Cells’ Survival and Hindering Cytochrome C-Mediated Apoptosis

**DOI:** 10.3390/ijms21197236

**Published:** 2020-09-30

**Authors:** Lana Nežić, Ranko Škrbić, Ljiljana Amidžić, Radoslav Gajanin, Zoran Milovanović, Eugenie Nepovimova, Kamil Kuča, Vesna Jaćević

**Affiliations:** 1 Department of Pharmacology, Toxicology and Clinical Pharmacology, School of Medicine, University of Banja Luka, 14 Save Mrkalja St, 78000 Banja Luka, Bosnia and Herzegovina; ranko.skrbic@med.unibl.org; 2 Center for Biomedical Research, School of Medicine, University of Banja Luka, 14 Save Mrkalja St, 78000 Banja Luka, Bosnia and Herzegovina; ljiljana.amidzic@med.unibl.org; 3 Institute of Pathology, University Clinical Center of Republic of Srpska, School of Medicine, University of Banja Luka, 12 Beba St, 78000 Banja Luka, Bosnia and Herzegovina; radoslav.gajanin@med.unibl.org; 4 Special Police Unit, Police Department of the City of Belgrade, Ministry of Interior, Trebevićka 12/A, 11030 Belgrade, Serbia; tinahoks41@gmail.com; 5 Department of Chemistry, Faculty of Science, University of Hradec Kralove, Rokitanského 62, 500 03 Hradec Králové, Czech Republic; evzenie.n@seznam.cz (E.N.); v_jacevic@yahoo.com (V.J.); 6 Biomedical Research Center, University Hospital Hradec Kralove, 500 02 Hradec Kralove, Czech Republic; 7 Department for Experimental Toxicology and Pharmacology, National Poison Control Centre, Military Medical Academy, 11 Crnotravska St, 11000 Belgrade, Serbia; 8 Department of Pharmacological Sciences, Medical Faculty of the Military Medical Academy, the University of Defence in Belgrade, 17 Crnotravska St, 11000 Belgrade, Serbia

**Keywords:** simvastatin, endotoxin, tubular apoptosis, cytochrome C, Bcl-XL, survivin

## Abstract

Increasing evidence suggests that apoptosis of tubular cells and renal inflammation mainly determine the outcome of sepsis-associated acute kidney injury (AKI). The study aim was to investigate the molecular mechanism involved in the renoprotective effects of simvastatin in endotoxin (lipopolysaccharide, LSP)-induced AKI. A sepsis model was established by intraperitoneal injection of a single non-lethal LPS dose after short-term simvastatin pretreatment. The severity of the inflammatory injury was expressed as renal damage scores (RDS). Apoptosis of tubular cells was detected by Terminal deoxynucleotidyl transferase-mediated dUTP Nick End Labeling (TUNEL assay) (apoptotic DNA fragmentation, expressed as an apoptotic index, AI) and immunohistochemical staining for cleaved caspase-3, cytochrome C, and anti-apoptotic Bcl-xL and survivin. We found that endotoxin induced severe renal inflammatory injury (RDS = 3.58 ± 0.50), whereas simvastatin dose-dependently prevented structural changes induced by LPS. Furthermore, simvastatin 40 mg/kg most profoundly attenuated tubular apoptosis, determined as a decrease of cytochrome C, caspase-3 expression, and AIs (*p*  <  0.01 vs. LPS). Conversely, simvastatin induced a significant increase of Bcl-XL and survivin, both in the strong inverse correlations with cleaved caspase-3 and cytochrome C. Our study indicates that simvastatin has cytoprotective effects against LPS-induced tubular apoptosis, seemingly mediated by upregulation of cell-survival molecules, such as Bcl-XL and survivin, and inhibition of the mitochondrial cytochrome C and downstream caspase-3 activation.

## 1. Introduction

Acute kidney injury (AKI) is one of the major complications of sepsis-induced multiple organ failure and is accompanied by high mortality [1]. During sepsis, a major mechanism of AKI includes severe inflammation in the renal parenchyma, heterogeneous distortion of microvascular flow at the peritubular and glomerular levels, and severe tubular epithelial injury and apoptosis [1,2].

Lipopolysaccharide (LPS), an endotoxin from the Gram-negative bacteria, has been identified as the major factor associated with the development of sepsis-associated AKI [3]. Proinflammatory cytokine, tumor necrosis factor-α (TNF-α), has a crucial role in the pathogenesis of the AKI caused by endotoxemia, leading to renal inflammatory injury, and acute tubular cell apoptosis presumably by activation of the extrinsic apoptotic pathway [1,4]. The pleiotropic cytokine TNF-α has a particular role in the initiation of cell-survival signaling molecules such as upregulation of anti-apoptotic molecules Bcl-2, and survivin (an inhibitor of apoptosis—IAP) through activation of nuclear factor-kappa (NF-κB) [5]. In addition to the severe inflammatory syndrome, LPS activates Toll-like receptor 4 (TLR4) that is present in the membrane of immune cells and renal tubular epithelial cells, triggering the excessive release of proinflammatory cytokines, oxidative stress, and tubular cell apoptosis [3,6]. Severe tubular cell apoptosis plays an important role in LPS-induced AKI, showing caspase-3-positivity in tubular epithelial cells even at the early phase of AKI [3,7].

Mitochondrial dysfunction and damage are confirmed in the LPS and cecal ligation puncture (CLP) model of septic AKI. Oxidative stress has also been known to contribute to the overproduction of mitochondrial reactive oxygen species (ROS) and early mitochondrial dysfunction after the LPS challenge, which triggers intrinsic apoptosis through the release of pro-apoptotic cytochrome C into the cytosol and activating the caspase cascade. Importantly, antiapoptotic Bcl-2 members, such as Bcl-XL, inhibit cell death by blocking of the cytochrome C release from mitochondria and thereby prevent downstream caspases’ (caspase-9, -3, -7) activation [1,2,8,9].

Consistent with these results, LPS-induced AKI and renal tubular cells apoptosis were ameliorated by novel potential agents, such as a pluripotent autocrine growth factor progranulin, an anion transporter uncoupling protein 2 [10], the bee venom [11], and peroxiredoxin protein DJ-1 (Parkinson disease protein 7, Park7) [12], as well as by vitamin D that suppressed p53-upregulated modulator of apoptosis (PUMA) and upregulated expression of a Bcl-2 family antiapoptotic protein [13]. Thus, an LPS-induced AKI model has been commonly recognized to study the pathophysiology of renal tissue injury and tubular cell apoptosis and to evaluate potential new therapeutic agents for this medical condition [2,3].

Numerous experimental studies demonstrated that statins, well-known lipid-lowering drugs, improved survival, and prevent tissue and organ injuries in local inflammation [14] or sepsis induced by LPS or CLP [15,16]. By inhibition of hydroxy-3-methylglutaryl-CoA (HMG-CoA), reductase statins block the mevalonate pathway and activation of intermediate products involved in cell signaling pathways, such as apoptosis or cell survival. One previous study showed that atorvastatin ameliorated contrast-induced nephropathy and reduced the extent of renal tubular cell apoptosis that is associated with the decreased expression of proapoptotic Bax/caspase-3 and increased Bcl-2 [17]. Another study that demonstrated the renoprotective effects of pitavastatin on cisplatin-induced AKI pointed to suppression of the mitogen-activated protein kinase (MAPK)/NF-kB/inflammation axis and intrinsic apoptotic pathway [18].

In the context of the experimental sepsis, we have previously shown that simvastatin improved survival rate and significantly suppressed LPS-induced over-production of proinflammatory cytokines, TNFα and interleukin (IL)-1β [19]. Furthermore, the cell-protective effect of simvastatin pretreatment against LPS has been confirmed on cardiomyocytes [20], hepatocytes, and spleen lymphocytes [21]. These results showed that pretreatment with simvastatin mitigated myocardial, liver, and spleen tissue injuries, and decreased activation of the cleaved caspase-3 along to the reduced apoptotic-cell death in the parenchyma. Simvastatin targets the cell-survival signaling pathway survivin/NF-κB/p65 and anti-apoptotic Bcl-XL in these cells, which appears a protective mechanism in response to LPS-induced tissue injury and programmed cell death [20,21].

Therefore, the present study was designed to determine whether pretreatment with simvastatin (1) ameliorates LPS-induced AKI, and, if it does, (2) to elucidate its role in hindering apoptotic death-inducing pathways in the tubular epithelial cells, and (3) subsequent upregulation of cell-survival mechanisms like survivin and Bcl-XL. 

## 2. Results

### 2.1. Protective Effects of Simvastatin on the LPS-Induced Acute Renal Injury

**Figure 1:**
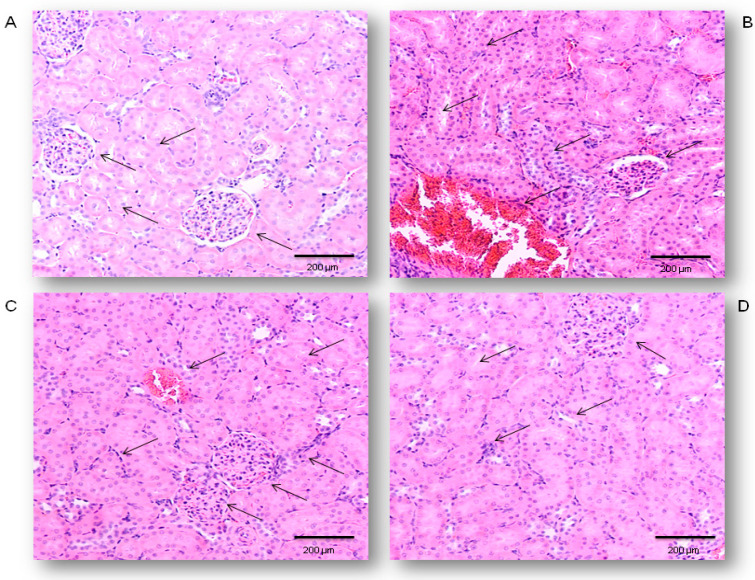
Protective effects of simvastatin pretreatment against LPS-induced acute renal damages, Hematoxylin and Eosin (H&E) method, 200× magnified images, black arrows indicates the gromeruls and tubules. (**A**) Appearance of renal tissue of control animals, (**B**) renal tissue challenged with LPS, (**C**) renal tissue from simvastatin 20 group, (**D**) renal tissue from simvastatin 40 group. Histopathological analysis revealed decreased renal inflammatory damages in both simvastatin-treated groups, while severe alterations persisted only in the LPS-treated group.

Renal specimens taken from the control rats revealed the normal histological structure of the glomeruli and renal tubules (Figure 1A). Treatment with LPS only (Figure 1B) induced congestion and small multifocal hemorrhages in the cortical and interstitial blood vessels with the presence of polymorphonuclear leukocytes (PMNL) infiltrate. Glomerular lesions were characterized by increased numbers of epithelial cells and pericapilar infiltration by PMNL and erythrocytes, predominantly. Both proximal and distal convoluted tubules showed diffuse epithelial cell swelling, loss of brush border, vacuolar degeneration, and focal necrosis. These changes were correlated with the RDS of 3.58 ± 0.50 confirming the LPS-induced severe renal damage (Table 1). Pretreatment with simvastatin 10 mg/kg reduced histopathological changes (RDS = 2.67  ± 0.48, not significantly vs. LPS group). Renal histopathological examination in the simvastatin 20 group (Figure 1C) showed the decreased intensity of tissue damage with the distinct renal tubular epithelial cell swelling and degeneration. Only individual glomeruli showed hypercellularity, with increased numbers of both resident cells and infiltrating leukocytes, indicative of the significantly lower RDS of 2.33  ± 0.47 (*p*  < 0.05 vs. control and vs. LPS group, respectively). Renal histology in the simvastatin 40 group was mostly unchanged and showed mild edema and hyperemia, rare small hemorrhages, and a single PNML infiltration throughout the cortex with irregular swelling of renal tubular cells. A mean RDS was minimal, 1.42 ± 0.50, in comparison to the LPS (*p* < 0.01). Semiquantitative assessment of renal tissue lesions reveals that simvastatin ameliorated LPS-induced histopathological changes in a dose-dependent manner (Figure 1C,D).

### 2.2. Simvastatin Inhibited Cleaved Caspase-3 Expression and Apoptotic Cell Death of Renal Tubular Epithelial Cells Induced by LPS

**Figure 2:**
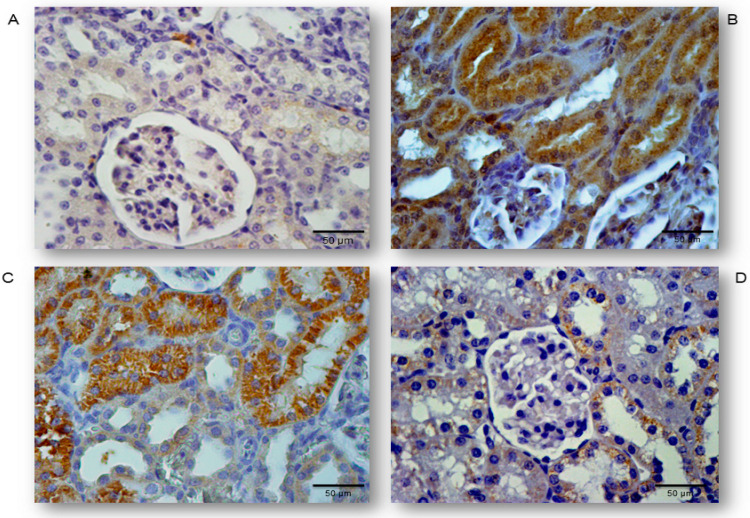
Representative images of apoptotic renal tubular epithelial cells that were challenged with LPS or pretreated with simvastatin prior to LPS, simvastatin 20 mg/kg or 40 mg/kg, respectively. Attenuation of the renal apoptosis induced by LPS shown as decreased cleaved caspase-3 expression in the tubular epithelial cells, assessed by immunohistochemistry, magnification 400×. (**A**) The control group, (**B**) intense cytoplasmic staining of cleaved caspase 3 in the tubular epithelium in the LPS group, as one of the feature of apoptotic cell, significant reduction of apoptotic cells in the groups treated with simvastatin 20 mg/kg (**C**) and 40 mg/kg, respectively (**D**).

**Figure 3:**
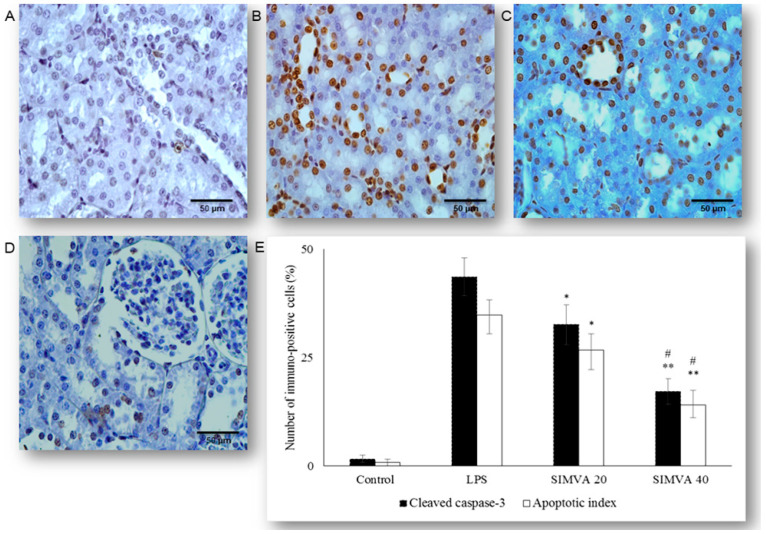
Simvastatin inhibited apoptosis of renal tubular epithelial cells in inflammatory injury induced by LPS, confirmed by TUNEL assay, magnification 400×. The apoptotic indices (AI) are based on the relative number of brown stained nuclei (TUNEL-positive renal tubular epithelial cells) and showed significant increase in the LPS group (**B**), simvastatin 20 mg/kg (**C**), and 40 mg/kg group (**D**) respectively, compared with the control group (**A**). LPS challenge led to a marked increase of TUNEL-positive tubular epithelial cells (shown as AIs in white columns AIs) while the AIs were significantly decreased in the simvastatin groups. Quantitative comparison of the immunohistochemically stained renal tissue for cleaved caspase 3 and the TUNEL-positive tubular epithelial cells expressed as AIs (* *p* <  0.05 vs. LPS group, ** *p*  <  0.05 vs. simvastatin 20 group, # *p*  <  0.01 vs. LPS group) (**E**).

Occurrence and the extent of apoptosis of the renal tubular epithelium was assessed based on the expression of cleaved caspase-3 and confirmed by the TUNEL assay with quantification of apoptotic index (AIs, see Methods and Materials Section) (Figure 2A–D and Figure 3A–D). We analyzed tissue sections challenged with simvastatin of 20 and 40 mg/kg, as the dose of 10 mg/kg did not show a significant protective effect on RDS. LPS induced cleavage of caspase-3 (active molecule) predominantly in the renal tubular epithelial cells, characteristically localized in the cytoplasm and perinuclear region of apoptotic cells. As shown in Figure 2, a substantial increase of cleaved caspase-3 expression in the LPS group (43.6% ± 4.4%, *p* < 0.01 vs. control group), was the most profoundly reduced with simvastatin 40 mg/kg (17.2% ± 2.9%, *p* < 0.01 vs. LPS group, and *p* < 0.05 vs. simvastatin 20 group), showing also and dose-dependent efficacy (Figure 2 and Figure 3E).

Definite apoptosis was confirmed with the TUNEL assay that detected chromatin condensation and DNA fragmentation, with the main features of apoptosis shown as dark brown nuclei (Figure 3). The apoptotic indices, as the degrees of apoptosis in renal tissue, significantly increased after LPS administration (in all experimental treated groups) compared with the control (*p* < 0.01), but they were markedly decreased in the group with simvastatin 20 (AI = 26.7% ;± 3.7%, *p* < 0.05) and simvastatin 40 group (AI = 14.0% ± 3.3%, *p* < 0.01) in respect to the LPS group (AI = 34.8% ± 3.6%) (Figure 3). Immunohistochemical staining and TUNEL assay revealed insignificant differences in the cleaved caspase-3 expression (total number of immuno-positive cells) compared to the AIs, that were strongly positively correlated across the experimental groups (*p* < 0.05) (Figure 3E). This could be explained by the fact that cytoplasmic immune-positivity of cleaved caspase-3 represents both apoptotic and the cells in pre-apoptosis without condensation of chromatin (TUNEL-positive cells) and with preserved cellular morphology.

### 2.3. Simvastatin Attenuated Expression of Pro-Apoptotic Cytochrome C in Renal Tubular Epithelial Cells after LPS Administration

**Figure 4:**
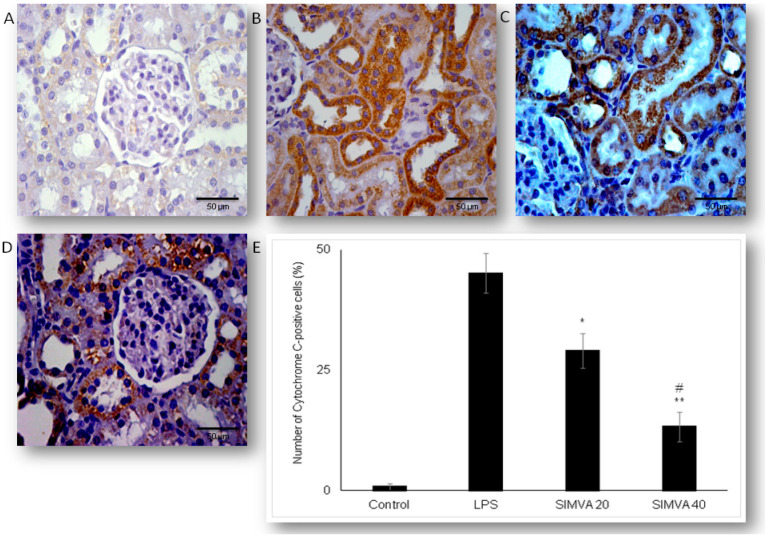
Simvastatin attenuated cytochrome C expression in renal tubular epithelial cells after LPS-administration. The expression of cytochrome C in rat renal tissue examined by immunohistochemical staining, magnification 400×, (**A**) negative immunostaining in the control group, (**B**) intense brown cytoplasmic staining in the tubular epithelium in the LPS group indicates increased expression of the pro-apoptotic protein cytochrome C. A significant decrease of cytochrome C-positive cells in the groups pretreated with simvastatin 20 mg/kg (**C**) and 40 mg/kg group (**D**), respectively. A quantitative analysis of cytochrome C-positive renal tubular epithelial cells assessed in the immunohistochemically stained sections in the renal tissue (* *p* < 0.05 vs. LPS group, ^∗∗^ *p* < 0.01 vs. simvastatin 20 group, # *p* < 0.01 vs. LPS group) (**E**).

To further investigate an apoptotic pathway targeted by simvastatin in LPS-induced AKI, we assessed mitochondrial pro-apoptotic marker, cytochrome C. The control group showed minimal immunostaining in the sporadic tubular epithelial cells. LPS administration increased expression of cytochrome C, quantified as the intense brown cytoplasmic staining in the affected tubular cells (45.11% ± 4.14%). Conversely, simvastatin markedly mitigated LPS-induced cytochrome C expression in comparison to the LPS group (Figure 4A–D). Consistently, quantitative analysis of cytochrome C immunopositivity showed a significant difference among groups (*p* ˂ 0.05 in simvastatin 20 group and *p* ˂ 0.01 in simvastatin 40 group vs. LPS, respectively), while very strong positive correlations between apoptotic markers, cleaved caspase-3, and cytochrome C were determined across the groups (*p* = 0.01).

### 2.4. Expression of Anti-Apoptotic Bcl-XL in Renal Tubular Epithelial Cells after Simvastatin and LPS Administration

**Figure 5:**
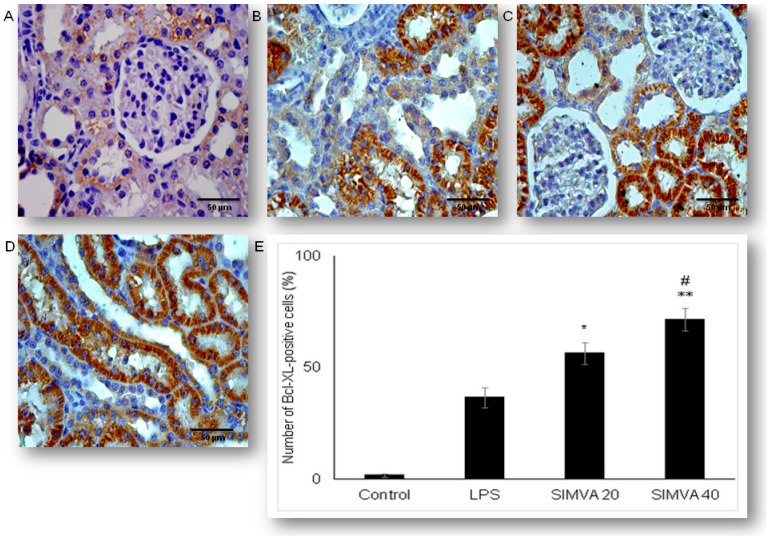
Simvastatin increased Bcl-XL expression in renal tubular epithelial cells after LPS administration. The expression of Bcl-XL in renal tissue was examined by immunohistochemical staining, magnification 400×, (**A**) the control group showed rare immuno-positive cells, (**B**) note Bcl-XL-expression in the tubular epithelial cells in the LPS group, in simvastatin 20 mg/kg (**C**) and 40 mg/kg group (**D**), respectively. Bcl-XL expression significantly increased, and it is determined as intensive brown cytoplasmic staining widely distributed in renal tubular epithelial cells. (**E**) A quantitative analysis of Bcl-XL-positive renal tubular epithelial cells assessed in immunohistochemically stained sections of the renal tissue (* *p* < 0.05 vs. LPS group, ^∗∗^ *p* < 0.05 vs. simvastatin 20 group, # *p* < 0.01 vs. LPS group).

**Figure 6:**
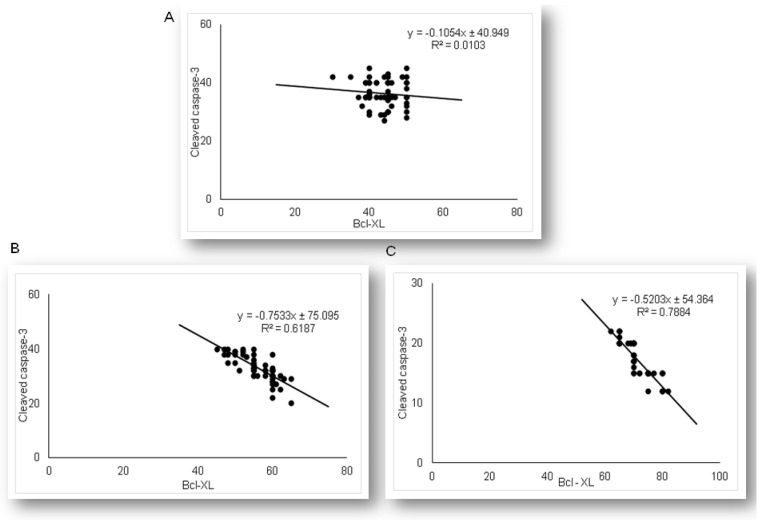
The correlations are shown for Bcl-XL and cleaved-caspase-3 in the renal tubular epithelial cells: (**A**) LPS group only, (**B**) simvastatin 20 mg/kg group, (**C**) simvastatin 40 mg/kg group.

**Figure 7:**
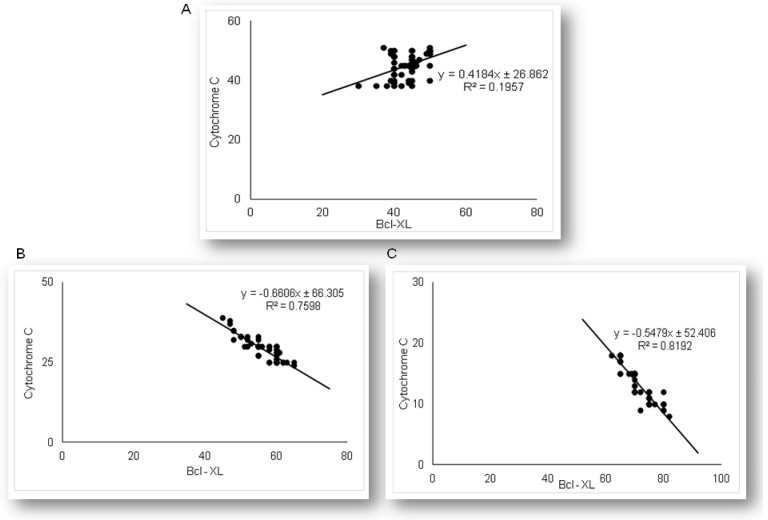
The correlations are shown for Bcl-XL and cytochrome C in the renal tubular epithelial cells: (**A**) LPS group only, (**B**) simvastatin 20 mg/kg group, (**C**) simvastatin 40 mg/kg group.

Expression of anti-apoptotic Bcl-xL in renal tubular epithelial cells significantly differed among LPS and simvastatin groups (Figure 5). Immuno-positive Bcl-XL renal tubular cells were sporadically determined in the control, and significantly in the LPS groups (36.4%, *p* < 0.05 vs. control) (Figure 5A,B). Upregulation of Bcl-XL implied that LPS might trigger a potential cell self-protective mechanism against the intrinsic apoptotic pathway. Pretreatment with simvastatin 20 mg/kg and simvastatin 40 mg/kg produced a gradual and significant increase in Bcl-xL expression in the tubular cells compared to the LPS group (56.4% ± 4.8%, *p* < 0.05 and 71.6% ± 4.9%, *p* < 0.01, respectively), showing intensive brown cytoplasmic staining (Figure 5A–E). To compare the expression of key apoptotic proteins and Bcl-XL in tubular cells in the treated groups, we analyzed their correlations (Figure 6). The result showed the strong inverse correlation between Bcl-XL and cleaved caspase-3-positive cells in the simvastatin 20 group (R^2^ = 0.61, *p* < 0.05) and simvastatin 40 group (R^2^ = 0.78, *p* < 0.05) group. As it is shown (Figure 7), Bcl-XL expression is in a very strong negative correlation with cytochrome C in the simvastatin 40 group (R^2^ = 0.81, *p* < 0.05), similarly as in the simvastatin 20 group, suggesting that through induction of anti-apoptotic Bcl-XL, simvastatin might express a cell-protective mechanism.

### 2.5. Simvastatin Enhanced Survivin Expression in Renal Tubular Epithelial Cells after LPS Administration

**Figure 8:**
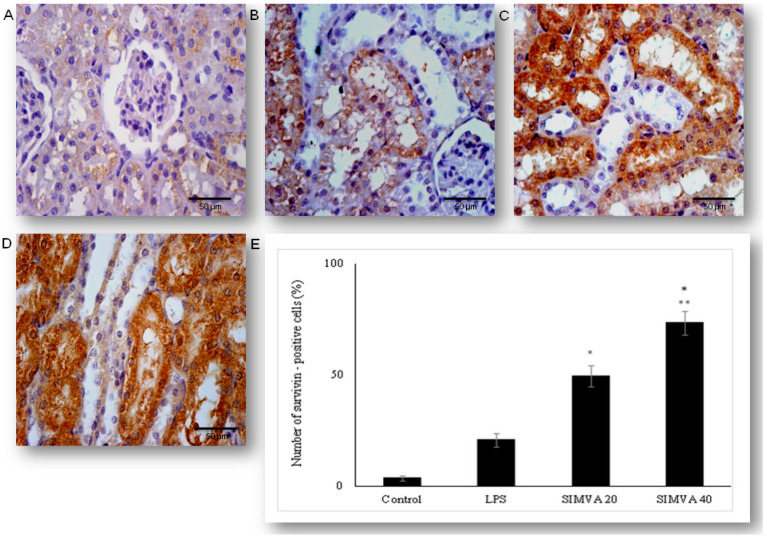
Simvastatin increased survivin expression in renal tubular epithelial cells after LPS administration. The expression of survivin in renal tissue examined by immunohistochemical staining, magnification 400×, (**A**) the control group showed rare immuno-positive cell, (**B**) note immuno-positive survivin cells in the LPS group, in simvastatin 20 group (**C**), and simvastatin 40 group (**D**), survivin expression significantly increased, and it is determined as intensive brown cytoplasmic staining widely distributed in renal tubular epithelial cells. (**E**) A quantitative analysis of survivin-positive renal tubular epithelial cells assessed in immunohistochemically stained sections of the renal tissue (* *p* < 0.01 vs. LPS group, ^∗∗^ *p* < 0.05 vs. simvastatin 20 group).

**Figure 9:**
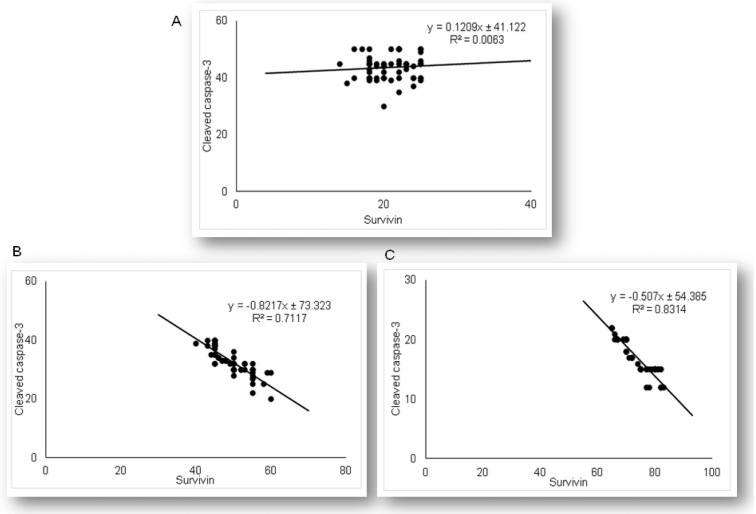
The correlations are shown for survivin and cleaved-caspase-3 in the renal tubular epithelial cells: (**A**) LPS group only, (**B**) simvastatin 20 mg/kg group, (**C**) simvastatin 40 mg/kg group.

**Figure 10:**
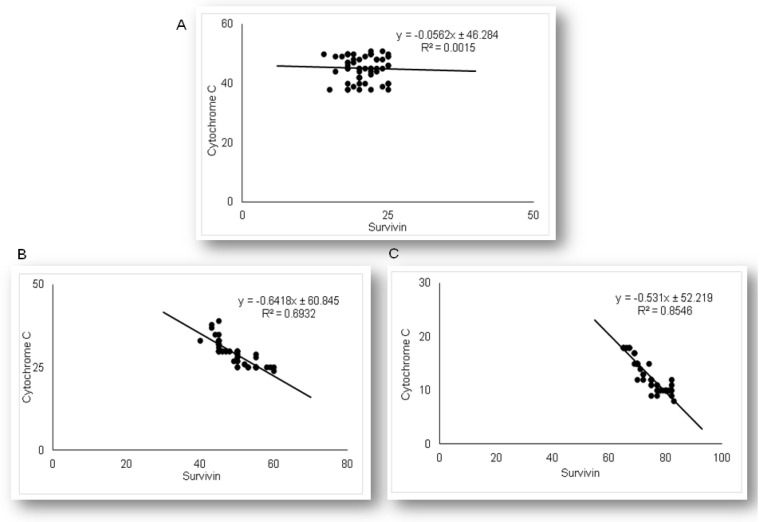
The correlations are shown for survivin and cytochrome C in the renal tubular epithelial cells: (**A**) LPS, (**B**) simvastatin 20 mg/kg group, (**C**) simvastatin 40 mg/kg group.

As the results demonstrated that simvastatin enhanced Bcl-XL expression in renal tubular epithelial cells after LPS administration, we further analyzed if simvastatin upregulates expression of a downstream inhibitor of apoptosis, survivin (Figure 8). Weak cytoplasmic staining assessed as positive survivin expression in the control was considered as its basal expression (Figure 8A). The LPS challenge resulted in a marked increase of survivin expression in tubular cells (*p* < 0.05 vs. control group), suggesting that survivin itself presents a cell-protective mechanism in LPS injury (Figure 8B). Further results demonstrated that simvastatin induced cell survival pathways, showing a dose-dependent increase in strong cytoplasmic expression of survivin (Figure 8C,D). Quantitative analysis revealed that pretreatment in simvastatin 20 and 40 groups led to a striking increase of survivin expression (49.5% ± 4.7% and 73.3% ± 5.3% vs. LPS group, *p* < 0.01, respectively) (Figure 8E). As survivin is one of the IAPs, we tested its correlation with cleaved caspase-3 and cytochrome C. As illustrated in Figure 9, strong inverse correlations were determined between survivin and cleaved caspase-3 in simvastatin 20 and 40 groups (R^2^ = 0.71 and R^2^ = 0.83, *p* < 0.01, respectively). Consistently, Pearson’s correlation analysis revealed a strong inverse correlation of survivin with cytochrome C-positive tubular cells in simvastatin 20 and 40 groups (R^2^ = 0.69 and R^2^ = 0.85, *p* < 0.01, respectively) (Figure 10), suggesting that simvastatin protects tubular cells in LPS-induced AKI by inhibiting key apoptotic proteins of the intrinsic pathway and activates cell-survival mechanisms.

## 3. Discussion

In the present study, a standard model of AKI induced by LPS was used to investigate the renoprotective effects of simvastatin on apoptotic signaling molecules in the development of AKI. The main findings indicate that without simvastatin, LPS severely damaged renal tissue, mainly due to induction of glomerular cell proliferation and tubular epithelial cell apoptosis mediated by a mitochondrial apoptotic pathway leading to caspase-3 cleavage. In contrast, with simvastatin pretreatment, the rats were protected against LPS-induced renal inflammatory injury, that is confirmed by attenuated apoptosis of tubular epithelial cells, and significantly increased expression of anti-apoptotic molecules, Bcl-XL and survivin. These results demonstrate that inhibition of cytochrome C apoptotic cascade and activation of IAP might be a mechanism of simvastatin cell-protective effects against bacterial toxin-associated AKI.

Growing experimental evidence has shown that pretreatment with statins prevents tissue injuries induced by bacterial toxins [20,21,22,23,24]. Consistently, our work and the studies by Apaya et al. [25] and Ozkok et al. [26] demonstrated that simvastatin attenuated LPS-induced renal injury, seen as a significantly reduced amount of infiltrating leukocytes associated with minimal histopathological features. It has been known that LPS/TLR4 signaling triggers systemic inflammation but also local renal inflammatory injury and apoptosis through the proinflammatory cytokines TNF-α release, to induce AKI [13]. Our previous results showed that simvastatin dose-dependently decreased overproduction of TNF-α and IL-1β in endotoxemia [19], therefore it is conceivable that in this study, simvastatin targets both inflammatory pathways to protect renal tubules against LPS.

Statins are known to have potent renoprotective effects in gentamicin-, cisplatin-, and cyclosporine-induced nephrotoxicity by a variety of mechanisms, ranging from antioxidant, anti-inflammatory, and anti-apoptotic effects [27]. However, to our knowledge, simvastatin suppression of tubular cell apoptosis in AKI associated with a septic condition has not been reported. Here, we showed that one of the potential protective mechanisms of simvastatin against AKI is blockade of renal tubular cell apoptosis, confirmed by reduced cytochrome C and cleaved caspase-3 expression and corresponding DNA fragmentation. Interestingly, results of antiapoptotic actions of simvastatin through inhibition of pro-apoptotic Bim/Bax and effectors caspases have been well documented in hepatocytes and lymphocytes, and cardiomyocyte in other’s and our previous studies [20,21,28,29].

Ultrastructural changes and dysfunctions of renal epithelial mitochondria appear to be an underlying mechanism in septic AKI, similarly to other sepsis-induced multi-organ failure [1,8]. A recent study by Liu et al. [9] showed dominant mitochondrial-mediated apoptosis in CLP-induced AKI with notable leakage of cytochrome C, followed by activation of downstream caspase-9 and -3, and disturbance of mitochondrial dynamics. In the present study, we observed that simvastatin abolished an LPS-induced significant increase of cytochrome C in renal tubular cells. This observation tightly correlates with a marked inhibition of cleaved caspase-3 in simvastatin groups, indicating its cell-protective mechanism against LPS.

Importantly, Bcl-2-related antiapoptotic protein such as Bcl-XL, control outer mitochondrial membrane integrity, bind to proapoptotic Bim/Bax proteins, and inhibit cell-death by preventing the release of pro-apoptotic factors such as cytochrome C or apoptosis-inducing factor [8,30]. Our results showed overexpression of Bcl-XL in tubular cells in simvastatin groups, accompanied by significantly decreased cytochrome C.

Consistently, our previous studies demonstrated that simvastatin upregulated Bcl-XL expression in LPS-challenged organs [20,21], while vitamin D or glycyrrhizin acid, an active ingredient of licorice, by targeting Bcl-2, suppressed tubular apoptosis, and markedly prevented LPS-induced AKI [13,31]. As Bcl-XL is one of the key anti-apoptotic proteins in renal tubular cells, our findings strongly suggest that simvastatin controls apoptosis by targeting Bcl-2 proteins and inhibiting cytochrome C.

Survivin, the unique member of the IAPs family, has a dual cellular role in the regulation of mitosis and inhibition of apoptosis. Biological functions of survivin depend on localization, so that in the cytosol (mitochondrial survivin), it initiates anti-apoptotic activity by blockade caspase cascade, while nuclear localization enables cell division. Cytoprotection by survivin is more selective and appears to target the cascade of mitochondrial cytochrome C-mediated apoptosis in order to prevent caspase-9 and downstream caspase-3 activation [5,32,33]. Previous studies have demonstrated that survivin, expressed dominantly in the proximal tubular cells, prevents development of AKI and renal apoptosis induced by various nephrotoxins (such as folic acid, cisplatin) by suppression of expression of the p53 gene [34,35] and in ischemia/reperfusion (I/R) injury through activation of the Notch-2 intracellular signaling pathway [36]. Our results have demonstrated increased cytoplasmic survivin expression in renal tubular cells in response to LPS that we assumed as induction of a cell-protection mechanism. Further, simvastatin induced intense and dose-dependent expression of survivin in the tubular epithelium that is inversely correlated with cytochrome C and cleaved caspase-3 respectively, and indicates its evident anti-apoptotic effects. Because our previous studies indicated an important role of survivin/NF-κB/p65 pathway activation in cytoprotection against LPS-injury [20,21], similar to Wilson et al. [37], in CLP-induced cardiomyopathy, we hypothesized that simvastatin has significant cell-protective effects in septic AKI but not only by inhibiting of apoptotic cell death but through induction of the important intracellular survival pathways in renal tubular epithelium.

## 4. Materials and Methods

### 4.1. Experimental Animals

Adult Wistar rats, 6–8 weeks old (200–220 g), raised at the Institute for Biomedical Researches, Military Medical Academy, Belgrade, Republic of Serbia, were used in this trial.

A typical macrolon plastic cage (Bioscape, Castrop-Rauxel, Germany) filled with clear sawdust (Versele-Laga, Deinze, Belgium) was used for experimental animals’ housing. Ambient conditions, the temperature of 22 ± 2 °C, the humidity of 55% ± 15%, air changes/h of 15–20, and the light/dark cycle of 12/12 h, in the animal housing room were centrally regulated. A commercial diet mixture for rats (Veterinary Institute Subotica, Subotica, Republic of Serbia) and tap water ad libitum were applied for animals’ feeding.

Before the start of the study, the experimental design, laboratory protocol, and welfare of the experimental animals were approved by the Ethics Committee of Experimental Animals of the Military Medical Academy, Belgrade, Serbia (No. 282-12/2002). This decision confirmed that in the complete experimental study, animal care and all treatments throughout the research are in compliance with Directive 2010/63/EU on the protection of animals used for scientific purposes and the Guidelines for Animal Welfare adopted by the Republic of Serbia (No. 323-07-04943/2014-05/1).

### 4.2. Drugs

The drug used in the experiment, simvastatin (donation for research purposes only, from pharmaceutical company Krka, Novo Mesto, Slovenia), was prepared in 0.5% methylcellulose as 10 or 20 mg/mL stocks.

Lipopolysaccharide (LPS, endotoxin, producer Sigma Aldrich, Munich, Germany), serotype 0127:B8 *Escherichia coli* was dissolved with sterile pyrogen-free physiologic saline and administered intraperitoneally (i.p.) immediately after dilution.

Each invasive procedure in animals was operated under aseptic conditions.

### 4.3. Experimental Design

In the model of experimental sepsis, we used endotoxin, and challenged the experimental animals with a non-lethal single dose of LPS i.p. (0.25 LD_50_/kg). This experimental sepsis is a widely accepted model that is featured with high-grade acute systemic inflammation, inflammatory infiltration, increased oxidative stress, and apoptosis of organ tissues [14,38,39]. Simvastatin was administered in the three different dose regimens (10, 20, and 40 mg/kg p.o.), that was confirmed in our previous experiments as the doses that completely protect the animals against the single median lethal dose (LD_50_) of 22.15 mg/kg i.p. of LPS in rats (95% CI 16.5–29.1) [19]. The dose selection of simvastatin was based on the previous rat/murine in vivo studies where the dose range was 10–100 mg/kg/day, considering a rapid upregulation (3- to 10-fold) of HMG-CoA reductase’s activity induced by statin treatment or other inhibitors in rodents. Therefore, the simvastatin doses in this experiment were higher compared to those recommended in clinical medicines [14,40,41].

A total of 30 animals were divided into five experimental groups and received the following treatments: (1) Control group (0.5% methylcellulose 1 mL/kg i.p.), (2) LPS group (non-lethal dose as 0.25 LD_50_/kg i.p., that is equal to 5.5 mg/kg of LPS i.p.), (3) Simvastatin 10 group (simvastatin 10 mg/kg p.o. + 0.25 LD_50_/kg LPS i.p.), (4) Simvastatin 20 group (simvastatin 20 mg/kg p.o. + 0.25 LD_50_/kg LPS i.p.), and (5) Simvastatin 40 group (simvastatin 40 mg/kg p.o. + 0.25 LD_50_/kg LPS i.p.).

After dissolution, simvastatin was given per os via oral gavage in the short-term treatment of 5 days, and the single non-lethal dose of LPS was administered 1.5 h after the simvastatin pretreatment. In the LPS group, animals received the same vehicle (1 mL/kg) of 0.5% methylcellulose for the same period as the simvastatin treatment, prior to LPS injection. The control group received an identical volume of vehicle only. After LPS administration, the animals were monitored continuously for 48 h and then sacrificed.

### 4.4. Histopathological Examination and Semiquantitative Analysis of Renal Damage Score

The renal protective effect of simvastatin was evaluated after receiving the last treatment. Before sacrification, all animals were immobilized in a dorsal position and euthanized by using sodium pentobarbital in a single dose of 30 mg/kg i.p. (Hemofarm AD, Vršac, Republic of Serbia). Shortly after the autopsy, a renal tissue sample from each animal was fixed in 10% neutral solution during the one-week period. Then, fixed renal tissue samples were divided into six equal sections, which were dehydrated in a series of alcohol (70%, 96%, and 100%) and xylene. After fitting into paraffin blocks, each 2 μm thick renal tissue section was stained using the hematoxylin and eosin (H&E) method.

The whole visual field from each renal slice was analyzed and photographed at magnification 200×, by using a light microscope connected with a digital camera (BX-45, Olympus, Tokyo, Japan) for histopathological examination, as per our method previously published in the literature [42,43,44,45,46,47,48]. To assess the degree of renal damages, which consists of edema, hyperemia, neutrophils infiltration, glomerular cells’ proliferation, and renal hemorrhages, a semi-quantitative 5-point scale was applied as previously described [43]. The severity of renal impairment expressed as Renal Damage Score (RDS) is shown in Table 2. The exact method for RDS calculation is presented in Table 1.

### 4.5. Detection and Quantification of Tubular Cell Apoptosis in Situ by TUNEL Method

The TUNEL (Terminal deoxynucleotidyl transferase-mediated dUTP Nick End Labeling) assay was used to assess the apoptosis of renal tubular cells. To perform TUNEL staining on paraffin-embedded sections (4–6 μm thickness), we used the In-Situ Cell Death Detection Kit POD (Roche Molecular Biochemicals, Basel, Switzerland, Cat. No 11 684 817 910) according to the manufacturer’s instructions. Renal tissue slides were incubated with anti-fluorescein antibody conjugated with horseradish peroxidase (POD), and then color development was performed using diaminobenzidine (DAB) substrate. According to these instructions, negative (incubation with Label Solution, instead of TUNEL reaction) and positive controls (incubation with DNase I recombinant, grade I) were performed.

Two blinded pathologists assessed TUNEL cells (immuno-positive reaction). The slides were examined under a light microscope (Olympus Plaza, Tokyo, Japan) at 400× magnifications. Twenty non-successive fields per sample were counted for the number of TUNEL-positive tubular cells. Apoptotic index (AI) defined as the percentage (%) of apoptotic tubular cells was calculated according to the formula (1):

(1) $$\mathrm{AI}\;\left(\%\;\mathrm{of}\;\mathrm{apoptotic}\;\mathrm{cells}\right)=\frac{\mathrm{the}\;\mathrm{number}\;\mathrm{of}\;\mathrm{TUNEL}-\mathrm{positive}\;\mathrm{tubular}\;\mathrm{cells}\;\times \;100}{\mathrm{total}\;\mathrm{number}\;\mathrm{of}\;\mathrm{tubular}\;\mathrm{cells}}$$

### 4.6. Detection and Quantification of Apoptosis Regulating Molecules by Immunohistochemistry

Paraffin-embedded sections of kidney tissues were stained with polyclonal rabbit antibodies for cleaved caspase-3 (Asp 175, Cat. 9661, Cell Signaling Technology, Frankfurt, Germany), monoclonal mouse antibody for Cytochrome C, clone 7H8.2C12 (Cat. MA5-11674, Invitrogen, Thermo Fisher Scientific, Walthman, MA, United States), polyclonal rabbit antibody for anti-apoptotic Bcl-XL (Cat. PA1-37161, Invitrogen, Thermo Fisher Scientific, Walthman, MA, United States), and monoclonal mouse antibody for survivin, clone 8E2 (Cat. MS-1201-P1 NeoMarkers Inc., Fremont, CA, The United States), according to the manufacturer’s instructions.

The standard protocol was followed for the immunohistochemistry staining on 3–4 μm deparaffinized and rehydrated tissue sections. Slides were then boiled for 20 min in a microwave oven with a citric acid buffer solution (0.01 mol/L citrate buffer, pH 6.0.). To reduce nonspecific background staining, slides were incubated in 3% hydrogen peroxide for 10 min. Primary antibodies for cleaved caspase-3 (1:300), Cytochrome C (1:100), Bcl-XL (RTU), and survivin (1:50) were applied according to the manufacturer’s recommended protocol. The slides were washed thoroughly with phosphate-buffered saline, pH 7.4, between the steps. 3,3′-Diaminobenzidine (DAB) (TL-015-HDJ, Thermo Scientific Lab Vision UltraVision ONE Detection System) was used as chromogen, to develop the antigen-antibody complex, and all slides were then counterstained with H&E, dehydrated, and mounted. Appropriate positive and negative controls were processed in parallel.

The slides were analyzed with a microscope (Olympus, Tokyo, Japan) at 400× magnification. For these sections, the average number of immune-positive cells (tubular cells with intensive optical density expression of cleaved caspase-3, Cytochrome C, BCL-XL, and survivin) across twenty non-successive fields was calculated by two independent pathologists in a blinded manner, using ImageJ software 1.50 (National Institute of Health, Bethesda, Rockville, MD, United States).

Quantitative measurement of the immuno-positive tubular cells was expressed according to this formula (2):

(2) $$\%\;\mathrm{of}\;\mathrm{positively}\;\mathrm{stained}\;\mathrm{cells}\;=\frac{\mathrm{the}\;\mathrm{number}\;\mathrm{of}\;\mathrm{positively}\;\mathrm{stained}\;\mathrm{tubular}\;\mathrm{cells}\;\times \;100}{\mathrm{total}\;\mathrm{number}\;\mathrm{of}\;\mathrm{tubular}\;\mathrm{cells}}$$

Survivin expression was evaluated qualitatively, where tubular cells positive for cytoplasmic staining were considered immuno-positive and taken into account [33,49,50].

### 4.7. Statistical Analysis

The statistical software package SPSS 19.0. (IBM Corporation, New York, NY, The United States) was used to analyze the results which were presented as mean value ($\bar{X}$) ± standard deviation (SD). To differentiate the renal damage score (RDS) as well as the expression of biomarkers among the groups, we used the Kruskal–Wallis rank test and analysis of variance (one-way ANOVA) followed by the Tamhane’s T2 post hoc test, respectively. Correlation analysis was presented as Pearson’s correlation coefficient. *p* < 0.05 was considered statistically significant.

## 5. Conclusions

This study broadened the current understanding of simvastatin anti-apoptotic and cytoprotective effects against LPS-induced AKI. Mechanistically, it seems that simvastatin inhibits the mitochondrial release of cytochrome C and consequent cleavage of the effector caspase-3, resulting in the blockade of tubular cells’ apoptosis. Moreover, simvastatin promotes cell-survival by enhancing Bcl-XL and survivin expression, signaling molecules are known that inhibit the intrinsic apoptotic pathway mediated by cytochrome C and directly block caspase-3 respectively, and in turn, inhibits apoptosis in tubular epithelium. Based on our findings in the AKI model reported here, we suggest that using simvastatin for the prevention of AKI in septic conditions is a promising therapeutic option in targeting apoptosis, and more preclinical and clinical studies should be encouraged in this regard.

## Figures and Tables

**Table 1:** The effects of different treatments on the degree of renal alterations—Renal Damage Score (RDS)

Treatment (mg/kg)	Renal Damage Score (6 Kidneys/Group × 6 Slices/Kidney)					$\bar{X}$ ± S.D.
	0	1	2	3	4	
Control	30	6	0	0	0	0.67 ± 0.48
LPS	0	0	0	15	21	3.58 ± 0.50 a^3^
Simvastatin 10 group	0	0	12	24	0	2.67 ± 0.48 a^1^
Simvastatin 20 group	0	0	24	12	0	2.33 ± 0.47 a^1^
Simvastatin 40 group	0	21	15	0	0	1.42 ± 0.50 b^2^

Statistical analysis was performed using the Kruskal–Wallis test. a^1^, a^3^—*p* < 0.05, 0.001 in comparison to the control group, b^2^—*p* < 0.01 in comparison to the LPS-only treated group. ($\bar{X}$—mean value, S.D—standard deviation).

**Table 2:** Tissue scoring scale for renal alterations—Renal Damage Score (RDS).

Degree	Description
0	Normal finding.
1	Mild damage: Single glomerular cells slightly enlarged. Mild dilatation of small blood vessels. A few foci of inflammatory cell infiltrates.
2	Moderate damage: < 50% glomerular cells with proliferation. Severe vasodilatation associated with hyperemia and edema. Various numbers of inflammatory cells infiltrates.
3	Severe and focal damage: > 50% glomerular cells with proliferation. Transmural rupture of the blood vessels (up to 50%) associated with an accumulation of inflammatory cells.
4	Severe and diffuse damage: Complete loss of the normal glomerular architecture, and the basal membrane and endothelial cells of the blood vessels (>50%). High-intensity hemorrhages and diffuse accumulation of inflammatory cells.

## References

[1] Gomez H., Kellum J.A. (2016). Sepsis-induced acute kidney injury. Curr. Opin. Crit. Care..

[2] Plotnikov E.Y., Brezgunova A.A., Pevzner I.B., Zorova L.D., Manskikh V.N., Popkov V.A., Silachev D.N., Zorov D.B. (2018). Mechanisms of LPS-Induced acute kidney injury in neonatal and adult rats. Antioxidants.

[3] Stasi A., Intini A., Divella C., Franzin R., Montemurno E., Grandaliano G., Ronco C., Fiaccadori E., Pertosa G.B., Gesualdo L. (2017). Emerging role of Lipopolysaccharide binding protein in sepsis-induced acute kidney injury. Nephrol. Dial. Transpl..

[4] Jo S.K., Cha D.R., Cho W.Y., Kim H.K., Chang K.H., Yun S.Y., Won N.H. (2002). Inflammatory cytokines and lipopolysaccharide induce Fas-mediated apoptosis in renal tubular cells. Nephron.

[5] Flusberg D.A., Sorger P.K. (2015). Surviving apoptosis: Life-death signalling in single cells. Trends Cell. Biol..

[6] Zhang S., Li R., Dong W., Yang H., Zhang L., Chen Y., Wang W., Li C., Wu Y., Ye Z. (2019). RIPK3 mediates renal tubular epithelial cell apoptosis in endotoxin-induced acute kidney injury. Mol. Med. Rep..

[7] Jacobs R., Honore P.M., Joannes-Boyau O., Boer W., De Regt J., De Waele E., Collin V., Spapen H.D. (2011). Septic acute kidney injury: The culprit is inflammatory apoptosis rather than ischemic necrosis. Blood Purif..

[8] Parikh S.M., Yang Y., He L., Tang C., Zhan M., Dong Z. (2015). Mitochondrial function and disturbances in the septic kidney. Semin. Nephrol..

[9] Liu J.X., Yang C., Zhang W.H., Su H.Y., Liu Z.J., Pan Q., Liu H.F. (2019). Disturbance of mitochondrial dynamics and mitophagy in sepsis-induced acute kidney injury. Life Sci..

[10] Zhong X., He J., Zhang X., Li C., Tian X., Xia W., Gan H., Xia Y. (2019). UCP2 alleviates tubular epithelial cell apoptosis in lipopolysaccharide-induced acute kidney injury by decreasing ROS production. Biomed. Pharm..

[11] Kim J.Y., Lee S.J., Maeng Y.I., Leem J., Park K.K. (2020). Protective Effects of Bee Venom against Endotoxemia-Related Acute Kidney Injury in Mice. Biology.

[12] Leeds J., Scindia Y., Loi V., Wlazlo E., Ghias E., Cechova S., Portilla D., Ledesma J., Swaminathan S. (2020). Protective Role of DJ-1 in Endotoxin-induced Acute Kidney Injury. Am. J. Physiol. Renal. Physiol.

[13] Du. J., Jiang S., Hu Z., Tang S., Sun Y., He J., Li Z., Yi B., Wang I., Zhang H. (2019). Vitamin D receptor activation protects against lipopolysaccharide-induced acute kidney injury through suppression of tubular cell apoptosis. Am. J. Physiol. Ren. Physiol..

[14] Nežić L., Škrbić R., Dobrić S., Stojiljković M.P., jaćevič V., Stoisavljević S., Milovanović Z.A., Stojaković N. (2009). Simvastatin and indomethacin have similar anti-inflammatory activity in a rat model of acute local inflammation. Basic Clin. Pharm. Toxicol..

[15] Zhao G., Yu Y.M., Kaneki M., Bonab A.A., Tompkins R.G., Fischman A.J. (2015). Simvastatin reduces burn injury-induced splenic apoptosis via down-regulation of the TNF-α/ NF-κB pathway. Ann. Surg..

[16] Wang Y., Yang W., Zhao X., Zhang R. (2018). Experimental study of the protective effect of simvastatin on lung injury in rats with sepsis. Inflammation.

[17] He X., Yang J., Li L., Tan H., Wu Y., Ran P., Sun S., Chen J., Zhou Y. (2017). Atorvastatin protects against contrast-induced nephropathy via anti-apoptosis by the upregulation of Hsp27 in vivo and in vitro. Mol. Med. Rep..

[18] Kaushik S., Tomar A., Puthanmadhom Narayanan S., Nag T.C., Arya D.C., Bhatia J. (2019). Pitavastatin attenuates cisplatin--induced renal injury by targeting MAPK and apoptotic pathways. J. Pharm. Pharm..

[19] Nežić L., Škrbić R., Dobrić S., Stojiljković M.P., Šatara S.S., Milovanović Z.A., Stojaković N. (2009). Effect of simvastatin on proinflammatory cytokines production during lipopolysaccharide-induced inflammation in rats. Gen. Physiol. Biophys..

[20] Nežić L., Škrbić R., Amidžić L.j., Gajanin R., Kuča K., Jaćević V. (2018). Simvastatin protects cardiomyocytes against endotoxin-induced apoptosis and up-regulates survivin/NF-κB/p65 expression. Sci. Rep..

[21] Nežić L., Amidžić L.j., Škrbić R., Gajanin R., Nepovimova E., Vališ M., Kuča K., Jaćević V. (2019). Simvastatin inhibits endotoxin-induced apoptosis in liver and spleen through up-regulation of survivin/NF-kB/p65 expression. Front. Pharm..

[22] Yasuda H., Yuen P.S., Hu X., Zhou H., Star R.A. (2006). Simvastatin improves sepsis-induced mortality and acute kidney injury via renal vascular effects. Kidn. Int..

[23] Chen C.H., Lee R.P., Wu W.T., Liao K.W., Hsu N., Hsu B.G. (2007). Fluvastatin ameliorates endotoxin-induced multiple organ failure in conscious rats. Resuscitation.

[24] Wang Y., Zhang L., Zhao X., Yang W., Zhang R. (2017). An experimental study of the protective effect of simvastatin on sepsis-induced myocardial depression in rats. Biomed. Pharm..

[25] Apaya M.K., Lin C.Y., Chiou C.Y., Yang C.C., Ting C.Y., Shyur L.F. (2016). Simvastatin and a plant galactolipid protect animals from septic shock by regulating oxylipin mediator dynamics through the MAPK-cPLA2 signalling pathway. Mol. Med..

[26] Özkök E., Yorulmaz H., Ateş G., Aydın I., Ergüven M., Tamer Ş. (2017). The impact of pretreatment with simvastatin on kidney tissue of rats with acute sepsis. Physiol. Int..

[27] Yang Y., Song M., Liu Y., Liu H., Sun L., Peng Y., Liu F., Venkatachalam M.A., Dong Z. (2016). Renoprotective approaches and strategies in acute kidney injury. Pharm. Ther..

[28] Shinozaki S., Inoue Y., Yang W., Fukaya M., Carter E.A., Yu Y., Fishman A., Tompkins R., Kanrki M. (2010). Farnesyltransferase inhibitor improved survival following endotoxin challenge in mice. Biochem. Biophys. Res. Commun..

[29] Slotta J.E., Laschke M.W., Schilling M.K., Menger M.D., Jeppsson B., Thorlacius H. (2010). Simvastatin attenuate hepatic sensitization to lipopolysaccharide after partial hepatectomy. J. Surg. Res..

[30] Lee E.F., Fairlie W.D. (2019). The Structural Biology of Bcl-xL. Int. J. Mol. Sci..

[31] Zhao H., Liu Z., Shen H., Jin S., Zhang S. (2016). Glycyrrhizic acid pretreatment prevents sepsis-induced acute kidney injury via suppressing inflammation, apoptosis and oxidative stress. Eur. J. Pharm..

[32] Dohi T., Beltrami E., Wall N.R., Plescia J., Altieri D.C. (2004). Mitochondrial survivin inhibits apoptosis and promotes tumorigenesis. J. Clin. Inv..

[33] Tsang T.J., Hsueh Y.C., Wei E.I., Lundy D.J., Cheng B., Chen Y.T., Wang S.S., Hsieh P.C.H. (2017). Subcellular localization of survivin determines its function in cardiomyocytes. Theranostics.

[34] Kindt N., Menzebach A., Van de Wouwer M., Betz I., De Vriese A., Conway E.M. (2008). Protective role of the inhibitor of apoptosis protein, survivin, in toxin-induced acute renal failure. Faseb. J..

[35] Yang C., Guo Y., Huang T.S., Zhao J., Huang X.J., Tang H.X., An N., Pan Q., Xu Y.-Z., Liu H.-F. (2018). Asiatic acid protects against cisplatin-induced acute kidney injury via anti-apoptosis and anti-inflammation. Biomed. Pharmacother..

[36] Chen J., Chen J.K., Conway E.M., Harris R.S. (2013). Survivin mediates renal proximal tubule recovery from AKI. J. Am. Soc. Nephrol..

[37] Wilson R.L., Selvaraju V., Lakshmanan R., Thirunavukkarasu M., Campbell J., McFadden D.W., Maulik D.W. (2017). Thioredoxin-1 attenuates sepsis-induced cardiomyopathy after cecal ligation and puncture in mice. J. Surg. Res..

[38] Seemann S., Zohles F., Lupp A. (2017). Comprehensive comparison of three different animal models for systemic inflammation. J. Biomed. Sci..

[39] Kosaka J., Lankadeva Y.R., May C.N., Bellomo R. (2016). Histopathology of septic acute kidney injury: A systematic review of experimental data. Crit. Care Med..

[40] Kita T., Brown M.S., Goldstein J.L. (1980). Feedback regulation of 3-hydroxy-3-methylglutaryl coenzyme A reductase in livers of mice treated with mevinolin, a competitive inhibitor of the reductase. J. Clin. Inv..

[41] Morel J., Hargreaves I., Brealey D., Neergheen V., Backman J.T., Lindig S., Bläss M., Bauer M., McAuley D.F., Singer M. (2017). Simvastatin pre-treatment improves survival and mitochondrial function in a 3-day fluid-resuscitated rat model of sepsis. Clin. Sci..

[42] Jaćević V., Jovic D., Kuča K., Dragojevic-Simic V., Dobric S., Trajkovic S., Borisev I., Segrt Z., Milovanovic Z., Bokonjic D. (2016). Effects of Fullerenol nanoparticles and Amifostine on radiation-induced tissue damages: Histopathological analysis. J. Appl. Biomed..

[43] Jaćević V., Djordjevic A., Srdjenovic B., Milic-Tores V., Segrt Z., Dragojevic-Simic V., Kuca K. (2017). Fullerenol nanoparticles prevent doxorubicin-induced acute hepatotoxicity in rats. Exp. Mol. Pathol..

[44] Jaćević V., Dragojević-Simić V., Tatomirović Ž., Dobrić S., Bokonjić D., Kovačević A., Nepovimova E., Vališ M., Kuča K. (2018). The efficacy of amifostine against multiple-dose doxorubicin-induced toxicity in rats. Int. J. Mol. Sci..

[45] Jaćević V., Nepovimova E., Kuča K. (2019). Toxic injury to the muscle tissue of rats following acute oximes exposure. Sci. Rep..

[46] Jaćević V., Nepovimova E., Kuča K. (2019). Acute toxic injuries of rat’s visceral tissues induced by different oximes. Sci. Rep..

[47] Jaćević V., Wu Q., Nepovimova E., Kuča K. (2019). Efficacy of methylprednisolone on T-2 toxin-induced cardiotoxicity in vivo: A pathohistological study. Environ. Toxicol. Pharm..

[48] Jaćević V., Wu Q., Nepovimova E., Kuča K. (2020). Cardiomiopythy induced by T-2 toxin. Food Chem. Toxicol..

[49] Wang K., Brems J.J., Gamelli R.L., Holterman X.A. (2010). Survivin signalling is regulated through the nuclear factor-kappa B pathway during glycochenodeoxycholate-induced hepatocyte apoptosis. Biochim. Biophys. Acta.

[50] Scheer A., Knauer S.K., Verhaegh R. (2017). Survivin expression pattern in the intestine of normoxic and ischemic rats. BMC Gastroenterol..

